# Epilithic *Chamaesiphon* (Synechococcales, Cyanobacteria) species in mountain streams of the Alps—interspecific differences in photo-physiological traits

**DOI:** 10.1007/s10811-017-1328-7

**Published:** 2017-11-04

**Authors:** Siegfried Aigner, Klaus Herburger, Andreas Holzinger, Ulf Karsten

**Affiliations:** 1 Institute of Botany, University of Innsbruck, Sternwarte Strasse 15, A-6020 Innsbruck, Austria; 2 Institute of Biological Sciences, University of Rostock, Albert-Einstein-Strasse 3, D-18057 Rostock, Germany

**Keywords:** Alpine stream, Light requirements, Photosynthesis, Pigments, Porphyra-334, Scytonemin

## Abstract

Many alpine streams inhabit conspicuous epilithic biofilms on pebbles and rocks that are formed by members of the cyanobacterial genus *Chamaesiphon* (Synechococcales). In the Austrian Alps, some *Chamaesiphon* species can even overgrow up to 70% of the surface of river rocks, and hence they must play an important but still unstudied ecological role in the organic matter flux. Since photo-biological traits have not been investigated so far, photosynthetic features, pigments, and UV-sunscreen compounds were studied in three *Chamaesiphon* morphospecies (*C. geitleri*, *C. polonicus*, *C. starmachii*). These species form conspicuously differently colored spots on cobbles and boulders in the alpine streams. While *C. polonicus* typically forms red crusts on flat pebble conglomerate, *C. geitleri* and *C. starmachii* are characterized by dark brown and black biofilms in the field, respectively. Photosynthesis-irradiance (PE) curves indicate that all three *Chamaesiphon* species have different light requirements for photosynthesis, with *C. starmachii* and *C. polonicus* preferring high and low photon fluence rates, respectively, while *C. geitleri* takes a position in between. This low-light requirement of *C. polonicus* is also reflected in ca. ten-times lower chlorophyll *a*, zeaxanthin, and ß-carotene concentrations, as well as in a lack of the UV-sunscreen scytonemin. All *Chamaesiphon* morphospecies exhibit the mycosporine-like amino acid porphyra-334. The physiological and biochemical data indicate strong intraspecific differences in photosynthetic activity and pigment patterns, which explain well the distinct preferences of the three studied *Chamaesiphon* morphospecies for sun-exposed or shaded habitats.

## Introduction

Members of the cyanobacterial genus *Chamaesiphon* (Synechococcales) typically form thin epilithic biofilms on stones and rocks in many mountain rivers all over the world (Rott et al. 2006; Rott 2008; Scott and Marcarelli 2012). In addition, *Chamaesiphon* species have been documented from rocks in streaming water in the Atlantic rainforest, Brazil (Sant’Anna et al. 2011), as epiphytes and epilithically growing in a fountain in Central Mexico (Gold-Morgan et al. 1996), in freshwaters of tropical North-Eastern Australia (McGregor 2013), and even from maritime Antarctica and Islands of North-Western Weddell Sea (Komárek 2014).

In the Austrian Alps, some *Chamaesiphon* species can cover even up to > 70% of the wetted surface of the available hard substrata in clear mountain streams (Rott and Wehr 2016). Although these biofilm communities have been so far neglected as a component of aquatic biota, they must play an important ecological role because of their conspicuous biomass, which points to some quantitative but still unstudied contribution in the organic matter flux (Rott and Wehr 2016).

From earlier publications, *Chamaesiphon* species were reported from habitats with distinct environmental settings. These include open rivers with full solar exposure and shaded streams, acidic to alkaline pH conditions, and ultra-oligotrophic to eutrophic nutrient concentrations (Cantonati et al. 2007; Rott and Schneider 2014). Due to the obviously different environmental requirements of various *Chamaesiphon* species, they are used for water quality assessment and bioindication in the frame of the so-called Periphyton Index of Trophic Status (PIT), which is a eutrophication metric for Nordic rivers (Schneider and Lindstrom 2011). In addition, Gutkowski et al. (2015) monitored between 2006 and 2012 > 400 sampling sites in soft-water streams in Germany and recorded 12 different *Chamaesiphon* species, of which the distribution was highly correlated with geochemical properties of the stream water followed by nutrient parameters.

Kann (1972) reported in her paper on the ecology of *Chamaesiphon* in the Swiss Alps that members of this genus preferentially grow in lotic (streaming) and lentic (eulitoral of lakes) waters, and that some species are desiccation-tolerant, at least temporarily, as well as eurytherm. In addition, Jaag (1945) described *Chamaesiphon polonicus* as primary settling phototrophic microorganism on rock walls moistened by melt water, which are fully exposed to solar radiation, pointing to high-light acclimation. So far, however, ecophysiological studies under controlled conditions are missing.

There are currently 92 *Chamaesiphon* species (and infraspecific) names in the database, of which 38 have been flagged as accepted taxonomically (http://www.algaebase.org, October 2017). *Chamaesiphon* species consist of unicellular, solitary, more or less elongated cells or groups of cells adhered to the hard substrate or forming microscopic shrub-like biofilms with densely and parallel gathered cells on stones, perpendicularly oriented to the substrate. The mountain stream taxa often cause visible colored spots on the stones (black, dark red), typically forming thin coatings < 5 mm (Rott and Wehr 2016).

More recently, Kurmayer et al. (2017) investigated for the first time three morphospecies of *Chamaesiphon* (*C. geitleri*, *C. polonicus*, *C. starmachii*) collected in alpine mountain streams of the Alps which were genetically analyzed for 16S rDNA nucleotide variability as well as studied concerning their ultrastructure. The phylogenetic results of these authors clearly confirmed the validity of the three morphospecies, but additionally indicated a high intraspecific genetic diversity among isolates of the same species, and the genus *Chamaesiphon* was not found to be monophyletic. Whether individual genotypes might exhibit strain-specific physiological traits is an open question for *Chamaesiphon* (Kurmayer et al. 2017). Ecotypic differentiation concerning light requirements has been documented for other aquatic cyanobacteria, for example, in *Synechococcus* spp. isolated from a microbial mat community of Mushroom Spring (Yellowstone National Park, Wyoming, USA) (Ferris et al. 2003). The designation of populations of a species as ecotypes that are locally adapted to specific environmental conditions (sensu Turesson 1922) remains, however, difficult, because of problems concerning species definition, which is particularly true in the Cyanobacteria (Albrecht et al. 2017). However, the presence of ecotypes as ecologically distinct units is accepted in order to characterize physiological diversity (Ferris et al. 2003).

In the present study, we studied for the first time various photo-biological traits of three *Chamaesiphon* species (*C. geitleri*, *C. polonicus*, *C. starmachii*) that inhabit different calcareous or siliceous mountain streams in the Tyrolean Alps (Austria) forming conspicuously differently colored spots on cobbles and boulders in these lotic ecosystems. While *C. polonicus* typically forms red crusts on flat pebble conglomerate, *C. geitleri* and *C. starmachii* are characterized by dark brown and black biofilms in the field, respectively. Based on this clearly distinguishable appearance and irradiation differences in the respective habitat (sun-exposed versus shade), various photo-biological traits of the three *Chamaesiphon* species were comparatively investigated. Besides photosynthetic features, pigments and UV-sunscreen compounds were studied. The data clearly indicates species-specific response patterns and biochemical profiles which well explains the respective occurrence in the different aquatic habitats.

## Material and methods

### Sampling area and studied *Chamaesiphon* species

The three studied *Chamaesiphon* species, *C. geitleri* Luther 1954, *C. polonicus* (Rostafinski) Hansgirg 1892, and *C. starmachii* Kann 1972, were collected on overgrown pebbles in two mountain streams of the Alps, Tyrol, Austria on 29 April 2016. *Chamaesiphon starmachii* originated from the Nederbach (Ochsengarten, near Kühtai), while *C. geitleri* and *C. polonicus* were sampled near the Isar spring. Habitat characteristics, abiotic factors, and water chemistry of both locations are given in Table 1.

**Table 1:** Abiotic factors and water chemistry of the habitats where the three morphospecies of *Chamaesiphon* (*C. geitleri*, *C. polonicus*, *C. starmachii*) were collected

Sampling site		
Parameter	Nederbach	Isar Spring
Species	*C. starmachii*	*C. geitleri*, *C. polonicus*
Coordinates	47° 13′ 45.16″ N	47° 23′ 01.86″ N
	10°57′28.98″E	11° 16′ 20.49″ E
Altitude a.s.l.	1593 m^*^	980 m
Temperature	10 °C	5 °C
pH	7.3	8.0
Light	Exposed	Shaded
Conductivity	46 μS cm^−1^	200 μS cm^−1^
Geochemistry	Siliceous	Calcareous
Flow velocity	n.d.	Min 0.4 m s^−1^
		Max 1.3 m s^−1^
		Maximum run-off 2.2 m^3^ s^−1^

*n.d.* not detected

^*^Next to this location is Lake Gossenkölle at 2400 m a.s.l., where up to 2069 μmol photons m^−2^ s^−1^ can be measured (Remias et al. 2010)

Subsurface water chemistry samples were taken simultaneously with the algal sampling. The nutrient analyses were performed at the AGES (Österreichische Agentur für Gesundheit und Ernährungssicherheit GmbH), Institute for Hydroanalytics, following Austrian standards related to Standard European methods (pH, ÖNORM EN ISO 10523; conductivity, ÖNORM EN 27888; NO_3_^−^-N, ÖNORM EN ISO 10304-1; NH_4_^+^, ÖNORM EN ISO 7150-1). Total and soluble reactive phosphorus were analyzed with a photometric method (Vogler 1966).

From the sampling sites, individual pebbles overgrown with the respective *Chamaesiphon* species were taken and transported into the laboratory under cold and dark conditions. Individual *Chamaesiphon* colonies were isolated under the dissecting and determined according to the morphospecies identification key of Komárek and Anagnostidis (1999). The cyanobacterial samples were further purified and separated from inorganic material using forceps (*C. starmachii*, *C. geitleri*) or Percoll-gradient centrifugation (*C. polonicus*; after Remias et al. 2012 using a modified 60/100%-percoll-gradient) leading to clonal material which was directly used for the photosynthetic measurements. For further biochemical analysis, the purified algal material was transferred onto GF/C-filters, immediately frozen in liquid nitrogen, lyophilized for 48 h, and stored at − 80 °C.

### Photosynthetic performance under light gradients

A PAM 2500 (Heinz Walz GmbH, Germany) was used to determine the effect of light gradients on the relative electron transport rates (rETRs) in *C. starmachii*, *C. geitleri*, and *C. polonicus* according to Herburger et al.(2015). Cyanobacterial samples were cut into small pieces with a razor blade and suspended in 0.6 mL of tap water, dark-adapted (15 min) in a KS-2500 suspension cuvette (Heinz Walz GmbH), and exposed to 8 (*C. starmachii*) or 6 (*C. geitleri*, *C. polonicus*) increasing light steps (each 30 s) up to 1000 μmol photons m^−2^ s^−1^. After each light step, the rETRs were calculated according to Schreiber and Bilger (1993), and photosynthesis-irradiance (PE) curve data were fitted according to Walsby (1994) or Webb et al. (1974), depending on whether photoinhibition occurred or not. Three photosynthetic parameters were derived from the photosynthetic models: linear curve increase at limiting photon fluence rates (α), maximum electron transport rate (rETR_max_), and the initial value of light-saturated photosynthesis (I_k_; μmol photons m^−2^ s^−1^).

### Biochemical analysis of photosynthetic pigments and UV-absorbing compounds

Freeze-dried material was ground with glass beads using a laboratory mill (Tissuelyser II, Qiagen, Venlo, the Netherlands) at 30 Hz for 10 min and extracted as described by Aigner et al. (2013). The powder was suspended in 1 mL methyl-tertbutylether (MTBE, Sigma-Aldrich, USA) containing 0.1% butylated hydroxytoluene (BHT, Sigma-Aldrich, USA) to prevent oxidation of pigments. Afterwards, extracts were vortexed and sonicated for 15 min at 0 °C, and the supernatants were removed whereby the ground material was again re-suspended in 1.5 mL MTBE to guarantee quantitative extraction. Both MTBE extracts were combined, and then 2 mL of 20% methanol (*v*/*v*; Roth, Germany) was added to the material, again shaken at 1200 rpm at 4 °C before the samples were frozen overnight at − 20 °C. These extracts were then centrifuged (1000×*g*, 5 min) at 4 °C supporting phase separation of the lipophilic supernatants (MTBE-phase) and the hydrophilic lower (methanol) phases. The upper and the lower phases were separated, evaporated to dryness in a SpeedVac (SPD111V, Thermo Fisher Scientific, USA), and then re-suspended in 150–350 μL N,N-dimethylformamide (DMF, Scharlau, Sentmenat, Spain; depending on the species) and 350 μL methanol (HPLC grade, Roth,, Germany), respectively. The extracts were centrifuged (15,000×*g*, 45 min, 4 °C) prior to injection into the HPLC.

Primary pigments were quantitatively analyzed according to Remias et al. (2005) and qualitatively identified after Remias and Lütz (2007), on an Agilent Technologies 1100 system (Germany), with a DAD-detector set at 440 nm for carotenoids and 662 nm for chlorophyll *a*. The column for quantitative analysis was a LiChroCART (C18, 100 × 4.6 mm, 5 μm, 120 Å) column (Agilent, Germany) at a flow rate of 1 mL min^−1^ using solvent A (acetonitrile to methanol = 74:6) and solvent B (methanol to hexane = 5:1). The system was started at 0% solvent B for 4 min, followed by a gradient to 100% solvent B from 4 to 9 min, which was maintained for 9 min, followed by a 5-min post-run with 100% solvent A. To analyze lipophilic pigments in greater detail, a qualitative analysis on the same system were applied using a LiChrospher RP C18 250 × 4 mm (Agilent) with a 4 × 4-mm pre-column of the same packing material with a flow rate of 1.4 mL^−1^ at 25 °C. The gradient was composed of 100% solvent A (acetonitrile to water to methanol to hexane = 80:7:3:1) from start to 15 min, followed by a linear gradient to 100% B from 15 to 32 min and ending at 45 min with 100% B; post-run 8 min 100% A. All solvents were of HPLC gradient grade quality. Pigment calibration and quantification was undertaken for ß-carotene and zeaxanthin with standards from Carbon 14 Centralen, Hørsholm, DK, while chlorophyll *a* was obtained from Sigma-Aldrich. All experimental manipulations were carried out at dim light and low temperatures.

The presence of secondary pigments (e.g., UV-absorbing compounds like MAAs) was analyzed from the hydrophilic phase on the same system and separated using a Supelcosil LC-NH2 column (RP18, 150 × 4.6 mm, 3 μm; Supelco, USA), protected with an RP18 guard cartridge (20 × 4.6 mm) of the same material at 30 °C at a flow rate of 1 mL min^−1^ after Aigner et al. (2017). Briefly, a gradient consisting of solvent A (0.1% ammonium formiate, pH 3.14) and solvent B (methanol) was used, starting with 75% solvent B, followed by a gradient to 30% solvent B within 5 min, then to 0% solvent B from 7 to 8 min, to 30% solvent B at 10 min, and then 75% solvent B at 15 min, followed by a 5 min post-run with 100% solvent A. Whole absorbance spectra were recorded each second at wavelengths between 310 and 330 nm. As MAA standard porphyra-334 isolated from the red alga, *Porphyra umbilicalis* collected from the rocky shore at the German North Sea Island Helgoland was used for quantification.

Extracellular substances (e.g., scytonemin) were extracted with ethanol (HPLC grade, Roth, Germany) according to Garcia-Pichel and Castenholz (1991), evaporated to dryness in a SpeedVac, re-suspended in methanol, and spectrophotometrically analyzed.

### Statistical analysis

PE curve-derived data (α, rETR_max_, I_k_; *n* = 4) and pigment data (*n* = 5) were compared by one-way ANOVA followed by Tukey’s post hoc test (*P* < 0.05) and subgroups of significantly different means were denoted.

## Results

### Habitat conditions

**Fig. 1:**
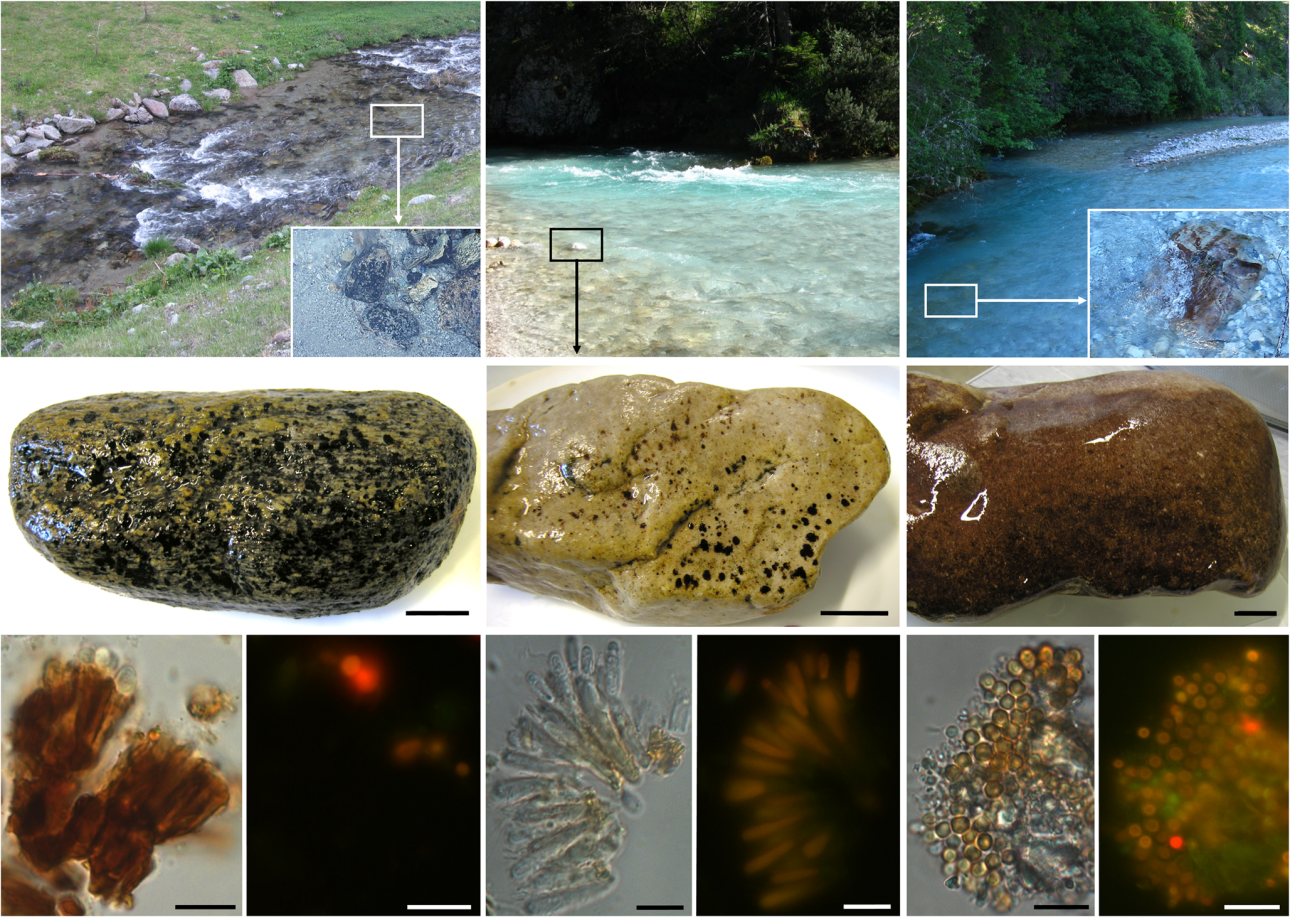
Representative photographs of three habitat sites (**a** Nederbach, exposed; **b** Isar spring, exposed; **c** Isar spring, shaded), where *Chamaesiphon starmachii* (**d**) *C. geitleri* (**e**), and *C. polonicus* (**f**), respectively, are abundant as epilithic biofilm spots on pebbles. Light microscopic and epifluorescence light microscopic pictures of *Chamaesiphon starmachii* (**g**, **h**), *C. geitleri* (**i**, **j**), and *C. polonicus* (**k**, **l**). Bars for pebbles 20 mm and for microscopic pictures 10 μm

The data in Tables 1 and 2 show some difference in the habitat preferences of the three *Chamaesiphon* morphospecies studied. The different habitats and macroscopic appearance of the morphospecies are visualized in Fig. 1. *C. starmachii* forms yellowish-brownish sheaths, and *C. polonicus* exhibits a marked reddish appearance (Fig. 1). *Chamaesiphon starmachii* prefers slightly lower pH values compared with *C. geitleri* and *C. polonicus*. On the other hand, *C. geitleri* exhibits a preference for extremely low nutrient concentrations as particularly reflected in low requirements for total phosphorus (TP, 9 μg L^−1^) and soluble reactive phosphorus (SRP, 4 μg L^−1^). In contrast, *C. polonicus* and *C. starmachii* prefer 17 and 24 μg L^−1^ TP, as well as 9 and 15 μg L^−1^ SRP, respectively (Table 2). While the requirement for nitrate is similar for all three *Chamaesiphon* morphospecies ranging from 510 to 660 μg L^−1^, *C. geitleri* prefers with 10 μg L^−1^ only half of the ammonia concentration of both other species (Table 2).

**Table 2:** Chemical habitat preferences of the three morphospecies of *Chamaesiphon* (*C. geitleri*, *C. polonicus*, *C. starmachii*) in Austrian mountain rivers (Rott and Wehr 2016)

Species	pH	Conductivity	TP	SRP	NH_4_^+^	NO_3_^−^
*C. starmachii*	7.93	209	24	15	25	660
*C. geitleri*	8.17	285	9	4	10	510
*C. polonicus*	8.06	303	17	9	20	620

All values represent the median (*n* > 45); conductivity is expressed as μS cm^−1^, nutrients are given as μg L^−1^

*TP* total phosphorus, *SRP* soluble reactive phosphorus

### Photosynthetic performance under light gradients

**Fig. 2:**
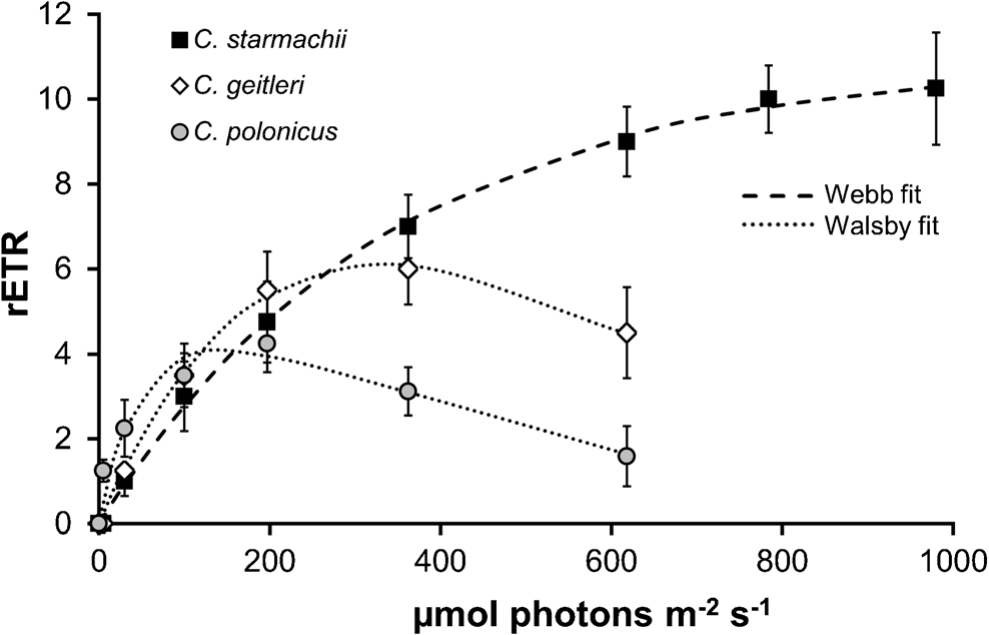
Photosynthesis-irradiance (PE) curves of all three morphospecies of *Chamaesiphon* (*C. geitleri*, *C. polonicus*, *C. starmachii*). Relative electron transport rates (rETR) were measured up to 1000 μmol photons m^−2^ s^−1^ and data points fitted with the photosynthetic model of Walsby (1997) (with photoinhibition) or Webb et al.(1974) (without photoinhibition)

**Fig. 3:**
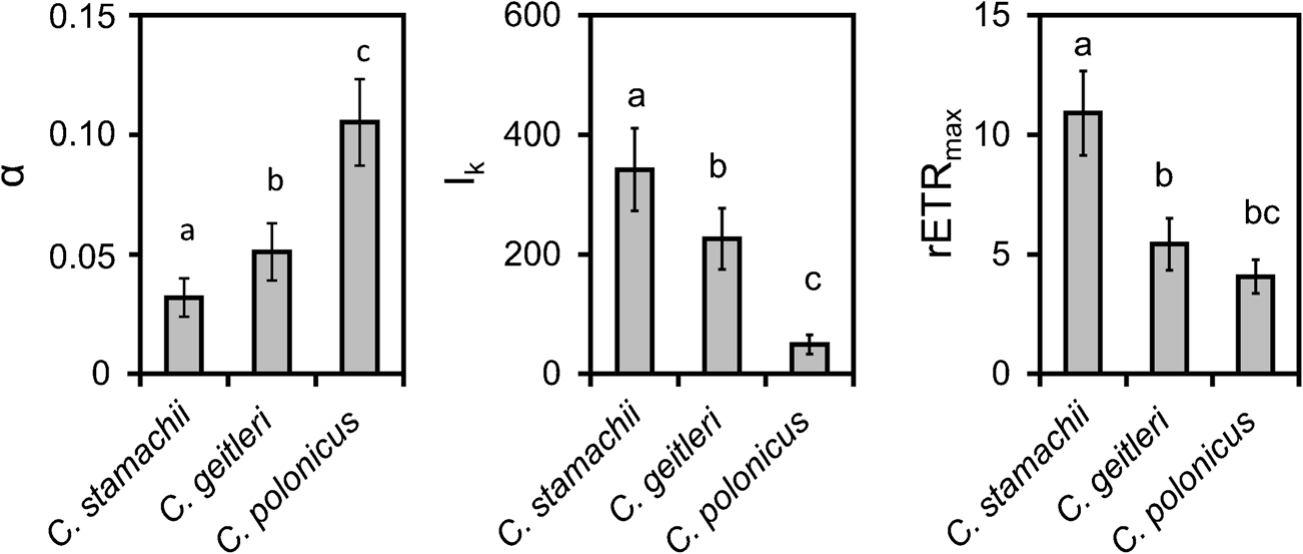
Comparison of the photosynthetic parameters derived from the rETR curves of the three morphospecies of *Chamaesiphon* (*C. geitleri*, *C. polonicus*, *C. starmachii*) (Fig. 2). Significantly different means between the cyanobacterial strains are indicated by small letters [α; electrons (μmol photons m^−2^ s^−1^)^−1^; rETR_max_, maximal relative electron transport rate; I_k_, μmol photons m^−2^ s^−1^]. Comparison was performed by one-way ANOVA followed by Tukey’s post hoc test (*P* < 0.05)

The three *Chamaesiphon* species investigated differed strongly in their photosynthetic response patterns to increasing photon fluence rates (Fig. 2), but they all exhibited continuously rising relative electron transport rates (rETR) up to ~ 200 μmol photons m^−2^ s^−1^. Further increase in the photon fluence rates led to an abrupt decrease of the rETR in *C. polonicus* reflecting strong photoinhibition. In contrast, the rETR of *C. geitleri* started declining above ~ 370 μmol photons m^−2^ s^−1^, while *C. starmachii* lacked photoinhibition, at least up to 1000 μmol photons m^−2^ s^−1^ (Fig. 2). The highest rETR_max_ value was measured in *C. starmachii* (Fig. 2). The species-specific PE curves coincided with a significantly higher photosynthetic performance under light-limited conditions (α) in *C. polonicus* compared to *C. geitleri* and *C. starmachii* (Fig. 3). In addition, the light saturation value (I_k_) was significantly higher in *C. starmachii*, followed by much lower values in *C. geitleri* and *C. polonicus*. The PE curve, α and I_k_ data indicate that all three *Chamaesiphon* species have remarkably different light requirements for photosynthesis, with *C. starmachii* and *C. polonicus* preferring high and low photon fluence rates, respectively, while *C. geitleri* takes a position in between (Fig. 3).

### Photosynthetic pigments and UV-absorbing compounds

*Chamaesiphon geitleri* and *C. starmachii* contained with 0.528 and 0.639 nmol chlorophyll *a* g^−1^ dry weight, respectively, at least ten-fold higher concentrations of this pigment compared with *C. polonicus* (0.052 nmol chlorophyll *a* g^−1^ dry weight) (Table 3). Similar differences between the three species were also detected for zeaxanthin and ß-carotene. However, *C. geitleri* exhibited with 0.416 nmol zeaxanthin g^−1^ dry weight and 0.267 nmol ß-carotene g^−1^ dry weight three-times more of the first and two-times more of the second pigment compared with *C. starmachii* (Table 3). *C. polonicus* contained less than 10% of these zeaxanthin and ß-carotene concentrations.

**Table 3:** Qualitative and quantitative composition of the most abundant primary pigments and mycosporine-like amino acid (MAA) porphyra-334 in the three morphospecies of *Chamaesiphon* (*C. geitleri*, *C. polonicus*, *C. starmachii*). Concentrations are given as nmol mg^−1^ dry weight for primary pigments and in μg mg^−1^ dry weight for porphyra-334 (*n* = 4 ± SD). For scytonemin, only its presence or lack could be documented

Species	Chlorophyll *a*	ß-Carotene	Zeaxanthin	Porphyra-334	Scytonemin
*C. starmachii*	0.639 ± 0.066^a^	0.132 ± 0.016^a^	0.139 ± 0.009^a^	1.651 ± 0.678^a^	+
*C. geitleri*	0.545 ± 0.289^a^	0.267 ± 0.019^b^	0.416 ± 0.009^b^	0.788 ± 0.146^a^	+
*C. polonicus*	0.052 ± 0.005^b^	0.011 ± 0.008^c^	0.017 ± 0.003^c^	0.064 ± 0.013^b^	–

Significant differences are depicted with different small letters (*P* < 0.05), “+” present, “−” lacking

**Fig. 4:**
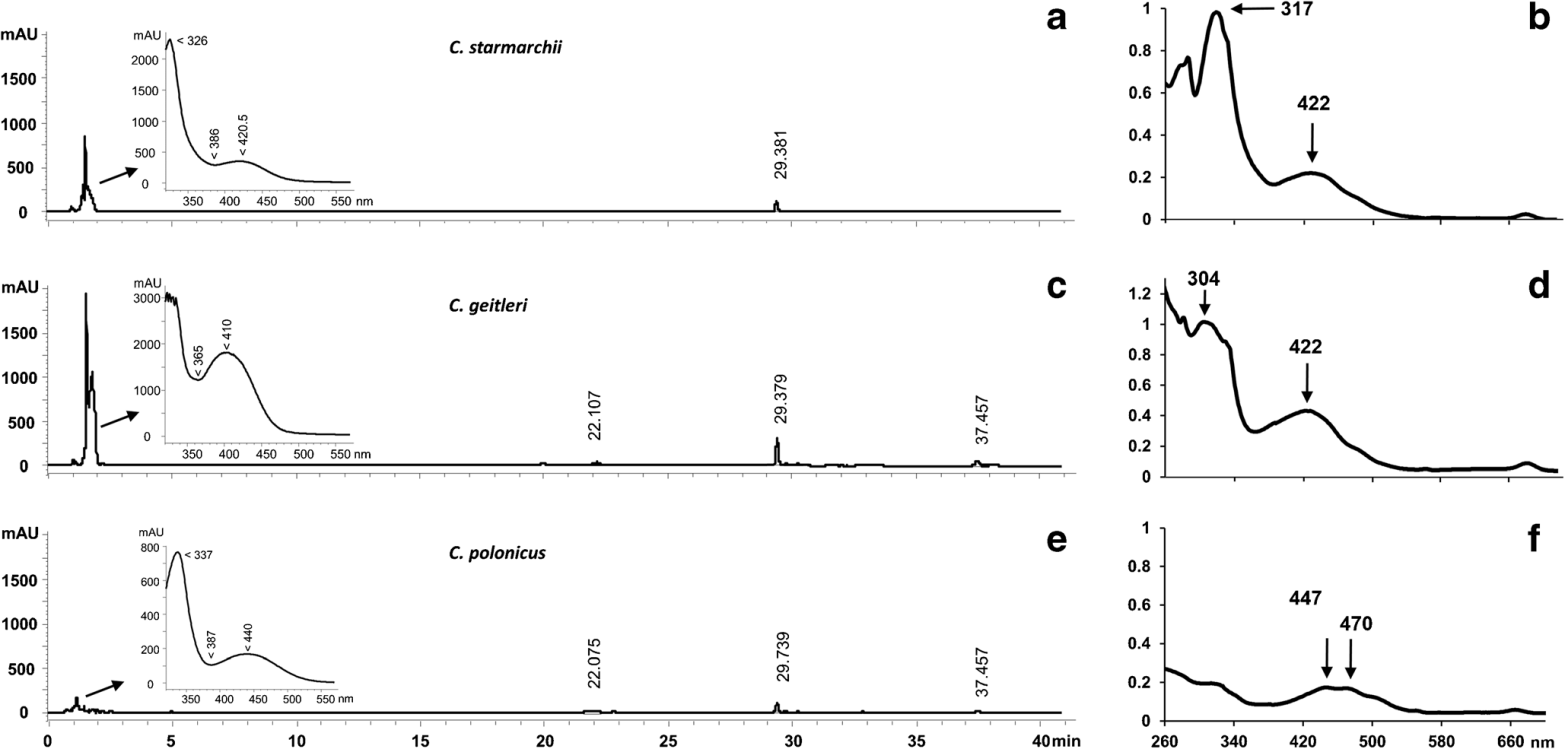
Comparison of lipid-soluble pigments of the three morphospecies of *Chamaesiphon* (*C. starmarchii*, *C. geitleri*, *C. polonicus*) during HPLC analysis. The chromatograms show the three major primary pigments zeaxanthin (22.1 min), chlorophyll *a* (29.3 and 29.7 min), and β-carotene (37.4 min) together with compounds of the extracellular sheath extracts (ESE). *Chamaesiphon starmarchii* and *C. geitleri* exhibit an early eluting peak (**a**, **c**) with a high absorption of the ESE in the UV-A and UV-B range (**b**, **d**), pointing to the presence of scytonemin. In *C. polonicus*, this substance is absent (**e**), and instead a compound with a carotene-like absorption is present in the ESE (**f**)

*Chamaesiphon starmarchii* and *C. geitleri* produce an extracelluarly located yellow-brown pigment as indicated by a brownish color of cell colonies under the microscope (Fig. 1). This brownish color is confirmed by a strong absorption in the UV-A range of sheath extracts (Fig. 4). In addition, during the pigment HPLC, the substance was detected as an early peak showing the characteristic absorption spectra for the well-described UV-sunscreen scytonemin (Fig. 4, Garcia-Pichel and Castenholz 1991). The data clearly indicate that *C. geitleri* and *C. starmachii* contain this pigment, whereby it is absent in *C. polonicus* (Table 3, Fig. 4). The latter species synthesizes instead a hydrophilic, orange carotene-like pigment with an absorption maximum at 470 nm, but lacking an additional absorption in the near UV-A range (Fig. 4).

The only UV-absorbing compound found in all three species was the MAA porphyra-334 (Table 3). The concentrations ranged from 0.06 μg porphyra-334 mg^−1^ dry weight in *C. polonicus* to 1.65 μg porphyra-334 mg^−1^ dry weight in *C. starmachii*, while *C. geitleri* showed with 0.42 μg porphyra-334 mg^−1^ dry weight a medium value (Table 3).

## Discussion

Cyanobacteria are abundant components of alpine freshwater ecosystems in temperate regions, particularly during mid- to late summer (Rott and Wehr 2016). The abundant occurrence of cyanobacteria at this time of the year has been partly attributed to their superior light-capturing capabilities when shading by shore vegetation and self-shading in colonies is the greatest (e.g., Tilzer 1987), and because of their high affinity for nitrogen and phosphorus when nutrient limitation is the most severe (Klemer and Konopka 1989). Although cyanobacteria are generally assumed to have high temperature optima for growth and photosynthesis (Robarts and Zohary 1987), many taxa represent the major component of autotrophic community biomass and productivity in polar lakes and streams, particularly in shallow water ecosystems (Vincent et al. 1992). The same is true for cold alpine streams (Rott and Wehr 2016).

Cyanobacteria are competitors for light due to their accessory pigmentation, i.e., phycobiliproteins and the structural organization of their light-harvesting antenna (Carey et al. 2012). Passarge et al. (2006) reported that the cyanobacterium *Synechocystis* sp. exploits attenuated light to lower levels than any other tested taxa, thereby out-competing other phytoplankton species when light was limiting.

In alpine streams, water chemistry, geochemical conditions, hydraulic conditions, and permanence of flow are the key factors defining the taxonomic diversity of cyanobacteria such as members of the abundant genus *Chamaesiphon* (Rott and Wehr 2016). Although *Chamaesiphon* species have been described as epiphytes and epilithic growing colonies on stones in many stream habitats of various alpine regions (Rott and Schneider 2014, Rott and Wehr 2016), only one specific study on their molecular phylogeny is existing (Kurmayer et al. 2017), and ecophysiological traits were not studied at all. A spatio-temporal analysis between 1989 and 1990 on field material of *C. geitleri* in an Austrian mountain stream well documented that the main biomass development occurred from late spring/early summer on when the water discharge decreased from 4–6 to 1–2 m^3^ s^−1^ (Rott and Wehr 2016). Closely connected to high flow volumes and velocities in mountain streams is typically the steady transport of sediment particles including pebbles, which leads to an unstable substratum for epilithic organisms. Consequently, large temporal water discharge can dramatically alter the physical habitat of such stream ecosystems, by movement and deposition of colonized stones, which strongly affect the biodiversity (Naiman et al. 2008). However, when spatial or temporal patterns in the flow regime are somewhat predictable, such as the annual spring snowmelt discharge peak in mountain streams, aquatic species such as *Chamaesiphon* have the opportunity to adapt and evolve strategies and responsive life histories to avoid or cope with, or even exploit these extreme hydrological events (Naiman et al. 2008; Rott and Wehr 2016).

In clear mountain streams, PAR and UV radiation (UVR) typically range from low levels in the presence of ice and snow cover during winter months to high levels during summer months, creating a need for protective mechanisms to survive. The Alps are one of the regions in Europe where the highest UVR levels occur (Schmucki and Philipona 2002). The altitude effect is depending on the wavelengths, i.e., ultraviolet-B (UV-B, 280–315 nm) is proportionally much stronger enhanced with increasing height than ultraviolet-A (UV-A, 315–400 nm) and PAR (Blumenthaler et al. 1996). In the present study, the photosynthetic activity measurements were based on the chlorophyll *a* fluorescence of photosystem II, which represent a broadly used approach in phycology (e.g., Schreiber 2004). The data indicates strong interspecific differences between the three *Chamaesiphon* morphospecies. While *C. starmachii* exhibited increasing rETRs with rising photon fluence rates without any indication of photoinhibition, at least up to 1000 μmol photons m^−2^ s^−1^ (Fig. 2), *C. geitleri* and *C. polonicus* showed strong photoinhibition already at moderate photon fluence rates between 200 and 370 μmol photons m^−2^ s^−1^. Different photosynthetic performances under light-limited conditions (α) and light saturation values (I_k_) (Fig. 3) suggest quite different light requirements for photosynthesis between the three morphospecies. While *C. starmachii* can be characterized as high-light acclimated, *C. polonicus* showed typical features of low-light acclimated organisms, and *C. geitleri* took a position between high- and low-light requirements. These physiological data are in accordance with the light regime at the collection site and the respective pigment concentrations. *Chamaesiphon geitleri* and *C. starmachii* contained ca. ten-times higher concentrations of chlorophyll *a*, zeaxanthin, and ß-carotene when compared with *C. polonicus* (Table 3). This observation is supported by the ultrastructural appearance of *Chamaesiphon* (Kurmayer et al. 2017). While *C. starmachii* and *C. geitleri* thylakoid membranes were clearly visible along the cell periphery by mostly irregular delimited bundles, in *C. polonicus* the thylakoid membranes were less pronounced and protruded across the cells. Moreover, the prominent occurrence of carboxysomes, which represent accumulations of ribulose-1,5-biphosphate carboxylase/oxygenase in *C. starmachii* and *C. geilteri* points towards a high carbon fixing activity (Kurmayer et al. 2017).

Since from alpine freshwater streams, only few data are available on light-preferences and UV-protection of Cyanobacteria; we compare the results of the present study with those of eukaryotic algae from alpine habitats. Two strains of the green alga *Zygogonium ericetorum* (Zygnematophyceae), which were collected from an alpine streamlet in the Alps at ~ 2300 m a.s.l. and from a Scottish Highland habitat, showed also PE curves that represent the habitat characteristics the algae were isolated from as well as whether vacuolar pigmentation (unusual phenolic compound) was present or not (Herburger et al. 2016). This corresponded to previous rETR measurements in a purple and green morph of *Z. ericetorum* isolated from the same alpine streamlet (Aigner et al. 2013). In addition, Aigner et al. (2013) stated that this Zygnematophycean green alga lacks UV-absorbing mycosporine-like amino acids or secondary carotenoids as expected for this class, making phenolic compounds particularity important for photoprotection. The abundant presence of these phenolic compounds preferentially in the purple morphs of *Z. ericetorum* was considered as a sunscreen and protector against harmful solar radiation.

In the case of *Chamaesiphon*, the qualitative pigment composition was identical in the three morphospecies, with only chlorophyll *a*, zeaxanthin, and ß-carotene present. This is not surprising as many cyanobacteria show a similar pattern (Kana et al. 1998; Takaichi 2011). For photosynthesis, both carotenoids and chlorophyll *a* are necessarily bound to peptides to form pigment-protein complexes in the thylakoid membrane. Phycobilisomes, light-harvesting antennae of photoysystem II, transfer the light via phycobiliproteins to chlorophyll *a*. In addition, in cyanobacteria, some carotenoids such as zeaxanthin and ß-carotene are located in the cytoplasmic membrane for protection from excessive light (Kana et al. 1998). β-Carotene is present in the reaction-center complexes (RC) and the light-harvesting complexes (LHC) of photosystem I (PSI) as well as the RC and the core LHC of photosystem II (PSII) (Takaichi 2011). Hence, particularly, β-carotene in both RC might have a photo-protective function, which seems to be preferentially expressed in *C. geitleri* and *C. starmachii*, while *C. polonicus* exhibited very low amounts. However, the macroscopic red appearance of this strain results most probably from dominance of phycobiliproteins, particularly of phycoerythrin over chlorophyll *a* (Bryant 1982), and the lack of scytonemin (see below).

Although many cyanobacteria have been reported to synthesize and accumulate UV-absorbing water-soluble MAAs that act as UV-sunscreen compounds (Garcia-Pichel and Castenholz 1993), this is the first report of porphyra-334 in members of the genus *Chamaesiphon* from alpine streams. While *C. starmachii* and *C. geitleri* contained with 1.65 and 0.42 μg porphyra-334 mg^−1^ dry weight, respectively, much higher MAA amounts compared to only 0.06 μg porphyra-334 mg^−1^ dry weight in *C. polonicus* (Table 3). The function of MAAs as UV-sunscreen has been experimentally proven in many aquatic organisms (e.g., Hartmann et al. 2015 and references therein), and hence it is reasonable to assume such a protective role also in *Chamaesiphon*. In addition, the presence of scytonemin in *C. geitleri* and *C. starmachii*, which is a dimeric indole-alkaloid and found exclusively among cyanobacteria, is also known as sunscreen compound (Garcia-Pichel and Castenholz 1991). This lipid-soluble pigment is typically located in the extracellular matrix of cyanobacterial cells and contributes to their brownish-yellow appearance. In fact, *C. geilteri* and *C. starmachii* showed yellowish-brownish-pigmented extracellular sheath coatings as described earlier (Kurmayer et al. 2017). Earlier data on microbial mats indicates the importance and effectiveness of scytonemin deposition in the outer sheaths of particularly upper-layer localized cyanobacteria as a sunscreen for the entire benthic community associated (Karsten et al. 1998). The sunscreen capacities of MAAs and scytonemin are higher if they are present simultaneously (Garcia-Pichel and Castenholz 1993), and therefore it is reasonable to assume that the sun-exposed species *C. geitleri* and *C. starmachii* use both compounds for photoprotection. Both species are forming black to brownish epilithic, coriaceous crusts, elevated from the stone, in contrast to *C. polonicus*, which appears as a reddish thin biofilm in the field preferring more shaded conditions. The appearance of scytonemin also correlates with morphological differences between these morphospecies. While *C. geitleri* and *C. starmachii* are producing sheaths (pseudovagina), enveloping a major or even complete part of the cells (Rott 2008), in *C. polonicus* such structures are absent (Fig. 1). The presence of scytonemin in the extracellular sheaths guarantees *C. geitleri* and *C. starmarchii* to retain this pigment in the colonies, thereby protecting lower cell layers and even dispersed cells after colony breakage due to hydromechanical forces in the alpine streams. Instead of scytonemin, *C. polonicus* is synthesizing a none-identified more hydrophilic, carotene-like substance with an absorption maximum at 470 nm without additional absorption in the upper UV-A range underpinning its shade adaptation.

Nutrient concentrations in mountain streams usually show large variations during the year with peaks in late winter and autumn, but mainly on a low concentration level (Rott and Wehr 2016). Although in many freshwater systems, phosphorus (P) is a limiting nutrient (Carey et al. 2012); cyanobacteria are known to overcome this limitation by various biochemical mechanisms including the ability to sequester intracellularly luxury P mainly as polyphosphate granula (Healey 1982). In fact, polyphosphate granules were found in the ultrastructure in all of the here investigated *Chamaesiphon* sp. (Kurmayer et al. 2017). As a result, cyanobacterial cells can theoretically double three to four times without having to uptake any additional P (Reynolds 2006), which provides a large competitive advantage in P-limiting environments such as mountain streams. The habitat preferences of the three *Chamaesiphon* morphospecies point to extremely low requirements for P (Table 2, Rott and Wehr 2016), which well explains their successful utilization for river water quality assessment in terms of the trophic status (Schneider and Lindstrom 2011).

In conclusion, members of the genus *Chamaesiphon* are abundant components in many mountain streams of the Alps where they form visible spots on pebbles. Intraspecific differences in photosynthetic activity as well as pigment and UV-sunscreen patterns well explain distinct preferences for sun-exposed or shaded habitats.

## References

[CR1] Aigner S, Remias D, Karsten U, Holzinger A (2013). Unusual phenolic compounds contribute to ecophysiological performance in the purple-colored green alga *Zygogonium ericetorum* (Zygnematophyceae Streptophyta) from a high-alpine habitat. J Phycol.

[CR2] Aigner S, Holzinger A, Karsten U, Kranner I (2017). The freshwater red alga *Batrachospermum turfosum* (Florideophyceae) can acclimate to a wide range of light and temperature conditions. Eur J Phycol.

[CR3] Albrecht M, Proeschold T, Schumann R (2017) Identification of cyanobacteria in a eutrophic coastal lagoon on the southern Baltic Coast. Front Microbiol doi. 10.3389/fmicb.2017.00923 10.3389/fmicb.2017.00923 PMC5446986 28611738

[CR4] Blumenthaler M, Ambach W, Möller R (1996). Increase in solar UV radiation with altitude. J Photochem Photobiol.

[CR5] Bryant DA (1982). Phycoerythrocyanin and phycoerythrin: properties and occurrence in cyanobacteria. Microbiol.

[CR6] Cantonati M, Rott E, Pfister P, Bertuzzi E, Cantonati M, Bertuzzi E, Spitale D (2007). Benthic algae in spring: biodiversity and sampling methods. The spring habitat: biota and sampling methods. Museo Tridentino di Scienze Naturali, Trento.

[CR7] Ferris MJ, Kuhl M, Wieland A, Ward DM (2003). Cyanobacterial ecotypes in different optical microenvironments of a 68 °C hot spring mat community revealed by 16S–23S rRNA internal transcribed spacer region variation. Appl Environ Microbiol.

[CR8] Garcia-Pichel F, Castenholz RW (1991). Characterization and biological implications of scytonemin, a cyanobacterial sheath pigment. J Phycol.

[CR9] Garcia-Pichel F, Castenholz RW (1993). Occurrence of UV-absorbing, mycosporine-like compounds among cyanobacterial isolates and an estimate of their screening capacity. Appl Environ Microbiol.

[CR10] Gold-Morgan M, Montejano G, Komárek J (1996). Freshwater epiphytic Chamaesiphonaceae from Central Mexico. Algol Stud.

[CR11] Gutkowski A, Foerster J, Doege A, Paul M (2015). *Chamaesiphon* species in soft-water streams in Germany: occurrence, ecology and use for bioindication. Algol Stud.

[CR12] Hartmann A, Becker K, Karsten U, Remias D, Ganzera M (2015). Analysis of mycosporine-like amino acids in selected algae and cyanobacteria by hydrophilic interaction liquid chromatography and a novel MAA from the red alga *Catenella repens*. Mar Drugs.

[CR13] Healey FP, Carr NG, Whitton BA (1982). Phosphate. The biology of cyanobacteria.

[CR14] Herburger K, Lewis LA, Holzinger A (2015). Photosynthetic efficiency, desiccation tolerance and ultrastructure in two phylogenetically distinct strains of alpine *Zygnema* sp. (Zygnematophyceae, Streptophyta): role of pre-akinete formation. Protoplasma.

[CR15] Herburger K, Remias D, Holzinger A (2016) The green alga *Zygogonium ericetorum* (Zygnematophyceae, Charophyta) shows high iron and aluminium tolerance: protection mechanisms and photosynthetic performance. FEMS Microbiol Ecol 92: doi. 10.1093/femsec/fiw103 10.1093/femsec/fiw103 PMC4909054 27178434

[CR16] Jaag O (1945). Untersuchungen über die Vegetation und Biologie der Algen des nackten Gesteins in den Alpen, im Jura und im Schweizerischen Mittelland. Beit Kryptogamenflora Schweiz.

[CR17] Kana TM, Glibert PM, Goericke R, Welschmeyer NA (1998). Zeaxanthin and β-carotene in *Synechococcus* WH7803 respond differently to irradiance. Limnol Oceanogr.

[CR18] Kann E (1972). Zur Systematik und Ökologie der Gattung *Chamaesiphon* (Cyanophyceae). 1. Systematik. Arch Hydrobiol Suppl.

[CR19] Karsten U, Maier J, Garcia-Pichel F (1998). Seasonality in UV-absorbing compounds of cyanobacterial mat communities from an intertidal mangrove flat. Aquat Microb Ecol.

[CR20] Klemer AR, Konopka AE (1989). Causes and consequences of blue-green algal (cyanobacterial) blooms. Lake Res Manag.

[CR21] Komárek J, Anagnostidis K (1999) Cyanoprokaryota, 1. Teil Chroococcales, vol 19/1. Gustav Fischer Verlag, Jena

[CR22] Komárek J (2014). Phenotypic and ecological diversity of freshwater coccoid cyanobacteria from maritime Antarctica and islands of NW Weddell Sea. II. Czech Polar Reports.

[CR23] Kurmayer R, Christiansen G, Holzinger A, Rott E (2017) Single colony genetic analysis of epilithic stream algae of the genus *Chamaesiphon* spp. Hydrobiologia. 10.1007/s10750-017-3295-z 10.1007/s10750-017-3295-z PMC5856356 29556110

[CR24] McGregor GB (2013). Freshwater cyanobacteria of north-eastern Australia: 2. Chroococcales. Phytotaxa.

[CR25] Naiman RJ, Latterell JJ, Pettit NE, Olden JD (2008). Flow variability and the biophysical vitality of river systems. Compt Rendus Geosci.

[CR26] Passarge J, Hol S, Escher M, Huisman J (2006). Competition for nutrients and light: stable coexistence, alternative stable states or competitive exclusion?. Ecol Monogr.

[CR27] Remias D, Lütz-Meindl U, Lütz C (2005). Photosynthesis, pigments and ultrastructure of the alpine snow alga *Chlamydomonas nivalis*. Eur J Phycol.

[CR28] Remias D, Karsten U, Lütz C, Leya T (2010). Physiological and morphological processes in the alpine snow alga *Chloromonas nivalis* (Chlorophyceae) during cyst formation. Protoplasma.

[CR29] Reynolds CS (2006). Ecology of phytoplankton.

[CR30] Robarts RD, Zoliary T (1987). Temperature effects on photosynthetic capacity, respiration arid growth rates of bloom-forming cyanobacteria. N Z J Mar Freshw Res.

[CR31] Rott E (2008). *Chamaesiphon komárekii* species nova, a new benthic freshwater chroococcalean species (Cyanophyta/Cyanobacteria) from western coniferous forest streams in British Columbia, Canada. Algol Stud.

[CR32] Rott E, Cantonati M, Füreder L, Pfister P (2006). Benthic algae in high altitude streams of the Alps—a neglected component of the aquatic biota. Hydrobiologia.

[CR33] Rott E, Schneider SC (2014). A comparison of ecological optima of soft-bodied benthic algae in Norwegian and Austrian rivers and consequences for river monitoring in Europe. Sci Total Environ.

[CR34] Rott E, Wehr JD, Necchi Jr O (2016). The spatio-temporal development of macroalgae in rivers. River Algae, Springer, Cham.

[CR35] Sant’Anna C, Gama W, Azevedo M, Komárek J (2011). New morphospecies of *Chamaesiphon* (Cyanobacteria) from Atlantic rainforest, Brazil. Fottea.

[CR36] Schmucki DA, Philipona R (2002). Ultraviolet radiation in the Alps: the altitude effect. Opt Eng.

[CR37] Schneider SC, Lindstrom EA (2011). The periphyton index of trophic status PIT: a new eutrophication metric based on non-diatomaceous benthic algae in Nordic rivers. Hydrobiologia.

[CR38] Schreiber U (2004) Pulse amplitude modulation (PAM) fluorometry and saturation pulse method: an overview. In: Papageorgiou GC (ed) Chlorophyll *a* fluorescence: a signature of photosynthesis, Kluwer Academic Press, Dordrecht, pp 279–319

[CR39] Schreiber U, Bilger W (1993). Progress in chlorophyll fluorescence research: major developments during the past years in retrospect. Progr Bot.

[CR40] Scott JT, Marcarelli AM, Whitton BA (2012). Cyanobacteria in freshwater benthic environments. Ecology of cyanobacteria II: their diversity in space and time.

[CR41] Takaichi S (2011). Carotenoids in algae: distributions, biosyntheses and functions. Mar Drugs.

[CR42] Tilzer MM (1987). Light-dependence of photosynthesis and growth in cyanobacteria: implications for their dominance in eutrophic lakes. N Z J Mar Freshw Biol.

[CR43] Turesson G (1922). The genotypical response of the plant species to the habitat. Hereditas.

[CR44] Vincent WF, Castenholz RW, Downes MT, Howard-Williams C (1992). Antarctic cyanobacteria: light, nutrients, and photosynthesis in the microbial mat environment. J Phycol.

[CR45] Vogler P (1966). Zur Analytik der Phosphorverbindungen in Gewässern. Limnologica.

[CR46] Walsby AE (1997). Numerical integration of phytoplankton photosynthesis through time and depth in a water column. New Phytol.

[CR47] Webb WL, Newton M, Starr D (1974). Carbon dioxide exchange of *Alnus rubra*: a mathematical model. Oecologia.

